# Weight change in chronic kidney disease: Association with mortality and kidney function

**DOI:** 10.1002/osp4.723

**Published:** 2023-11-22

**Authors:** Richard Singer, Hsin‐Chia Huang

**Affiliations:** ^1^ Canberra Health Services Renal Unit Garran Australian Capital Territory Australia; ^2^ School of Medicine Australian National University Acton Australian Capital Territory Australia; ^3^ Canberra Health Services, Respiratory and Sleep Medicine Garran Australian Capital Territory Australia

**Keywords:** body mass index, body weight change, mortality, obese, renal insufficiency

## Abstract

**Background:**

Excess body weight is a risk factor for the progression of chronic kidney disease (CKD), but weight loss in CKD has been associated with higher mortality. Consequently, blanket weight loss recommendations in this population are controversial. Little data is available on the patterns of weight‐change in CKD. The authors aimed to describe weight‐changes in moderate/severe CKD and explore associations with mortality and renal endpoints in patients with overweight and obesity.

**Methods:**

Non‐dialysis Canberra Hospital patients with estimated glomerular filtration (eGFR) < 60 mL/min/1.73 m^2^ and body mass index (BMI) ≥25 kg/m^2^ were followed for up to 5.5 years. Weight‐change ≥5% was considered clinically significant. The renal endpoint was defined as the commencement of dialysis or transplant or a ≥40% fall in eGFR. Relationships between weight‐change in the first year of follow‐up and mortality or the renal endpoint were assessed using Cox‐regression.

**Results:**

Three hundred ten patients (median age 75, median BMI 31 kg/m^2^) were identified. 68% had Stage‐4 CKD at baseline. Over 4.4‐years median follow‐up, 128 died and 140 had significant weight‐change. During the first year of follow‐up, 42 patients lost and 23 gained ≥5% body weight, of whom only 3 had intentionally lost weight. On multivariate regression, significant weight loss/gain at 1‐year was associated with 2.74 (*p* < 0.0005) and 2.67 (*p* = 0.003) hazard of subsequent death and with 2.51 (*p* = 0.004) and 2.20 (*p* = 0.05) hazard of the renal endpoint respectively. There was no association between baseline eGFR and subsequent weight change.

**Conclusions:**

Patients with moderate/severe CKD experience significant weight‐change, but this has no relationship to baseline kidney function. Significant weight‐change is associated with higher subsequent mortality and loss of kidney function, but this association is likely significantly affected by confounding.

## Graphical abstract

Clinically significant weight change is common in moderate to severe chronic kidney disease but has no relationship to baseline kidney function. Clinically significant weight change in those with overweight or obesity and chronic kidney disease is associated with higher mortality and worse renal outcomes, but this relationship is likely due to confounding.

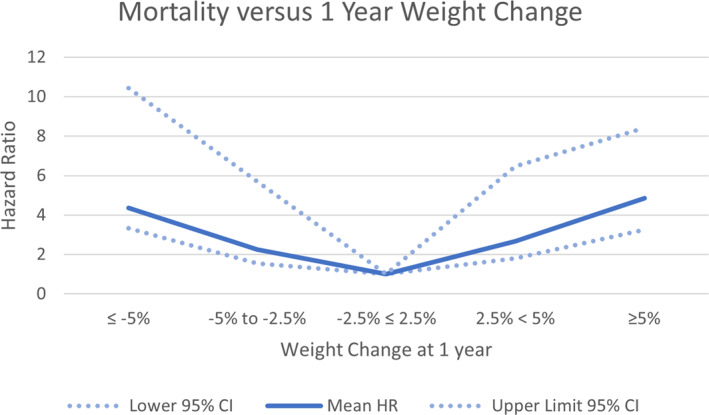

## 1 INTRODUCTION

Obesity is a risk factor for chronic kidney disease (CKD).^1^ Other risk factors for CKD, such as hypertension or proteinuria are improved by intentional weight loss^2^ and weight loss is recommended in those with overweight or obesity, especially in the presence of CKD.^3^ Excess body weight results in glomerular hyperfiltration.^4^ This can lead directly to albuminuria and to progressive CKD from the specific obesity related glomerular lesion of secondary focal and segmental glomerulosclerosis.^5^ However, obesity also accelerates the loss of kidney function in patients with unrelated causes of CKD, such as glomerulonephritis.^6^ In patients with obesity, weight loss resulting from metabolic bariatric surgery is associated with improvement in hypertension and substantially reduced mortality.^7^, ^8^ Mortality benefit has also been shown in dialysis patients who undergo metabolic bariatric surgery.^9^ However, other observational studies in CKD indicate that a higher body mass index (BMI) reduces mortality and that weight loss prior to dialysis increases mortality.^10^, ^11^, ^12^ Reasons for this are unclear but may be due to confounding, as a delay initiating dialysis in severe kidney failure or development of significant intercurrent illness can cause weight loss from loss of appetite or weight gain from fluid retention.

There is limited information published on the usual pattern of weight change in those with moderate to severe kidney disease and elevated BMI. Data that are available, such as by Harhay et al., are difficult to use clinically as the complex weight trajectory curves described can only be determined after several years of observation.^13^ A study published earlier that, in part, used data from Harhay's cohort, reported information on annualized weight change, with outcomes reported only in the subset of CKD patients who went on to commence dialysis during study follow‐up.^11^ Annualized weight changes are highly dependent on the follow‐up time, as small changes over a short period of time become relatively large changes if extrapolated over a year. In addition, clinicians do not know ahead of time when or whether their patients will start dialysis. This makes it difficult to use much of the published data for risk assessment in CKD until after dialysis has commenced. Published data in a Korean cohort with BMI <25 kg/m^2^ and CKD found a U‐shaped relationship with both significant weight gain and weight loss being associated with worse outcome.^12^

The Canberra Hospital Renal Unit provides tertiary referral public nephrology care for Canberra and the surrounding New South Wales (NSW) region. It serves a population of approximately 600,000 with free at the point of care public clinics conducted in Canberra and surrounding locations in NSW. The primary aim of this study was to describe the pattern of weight changes in patients with moderate to severe CKD who also had overweight or obesity. The secondary aims were to explore associations between changes in weight during the first year of follow‐up, subsequent mortality and subsequent loss of kidney function.

Ethical approval for this study was provided by the Australian Capital Territory (ACT) Health Human Research Ethics Committee (2022/ETH02130).

## 2 MATERIALS AND METHODS

The electronic medical record was searched to identify patients with estimated glomerular filtration (eGFR) < 60 mL/min/1.73 m^2^. Inclusion criteria were patients receiving nephrology care from the Canberra Hospital Renal Unit on 20 March 2017, with BMI≥25 kg/m^2^ and Stage 3, 4 or 5 CKD. The stages of CKD were defined as; stage 3 (eGFR 30–60), stage 4 (eGFR 15–30) and stage 5 (eGFR <15).^14^ Patient eGFR was calculated using the CKD‐EPI equation,^15^ without correction for race. Patients receiving renal replacement therapy (dialysis or kidney transplant) at baseline were excluded.

The electronic medical record contained data on sequential body weight, blood pressure, urine protein and eGFR at varying intervals. BP, weight and eGFR data closest to each 6‐month time point after 20 March 2017 were considered valid if collected no more than 180 days prior to a given time point. These data were recorded as missing if their date of collection was more than 180 days prior to the time point. Blood pressure, weight and eGFR data collected after a given time point were not used, except for month 60 data, where data were included if collected up to 30 September 2022.

Baseline proteinuria was included if collected within 180 days of 20 March 2017. Urine protein was available as either a dipstick result, a urine albumin/creatinine ratio, or a urine protein/creatinine ratio. Stage 3 albuminuria was considered to be present if there was a urine dipstick of ≥300 mg/L, a urine albumin/creatinine >30 mg/mmol, or a urine protein/creatinine ratio >76 mg/mmol.^16^ The cause of kidney failure was that stated by the treating physician or when not stated, was determined by one of the authors (RS) from a review of the medical record.

Patients were followed up until September 30, 2022. Renal replacement therapy initiation has significant effects on body weight and calculated eGFR; therefore, these variables were not analyzed after a patient initiated renal replacement therapy. A clinically significant fall in kidney function over a follow‐up (the renal endpoint) was considered to have occurred if there was either a ≥40% decline in eGFR that was maintained on subsequent assessments or if the patient commenced renal replacement therapy. It was considered very likely that patients with stage 5 CKD at baseline would either die, or commence dialysis during follow‐up, irrespective of any weight changes, so they were excluded from regression analysis for the renal endpoint.

The percentage weight change at 1 year was calculated by subtracting the baseline weight from the weight recorded closest to 1 year, dividing by baseline weight and multiplying by 100. A weight change of ≥5% from baseline was considered clinically significant. The main cause of clinically significant weight change at 1 year was determined by reviewing the clinical records. Where a cause was not stated in the record and if no cause was apparent from a review of the record, it was listed as “unknown”. Clinically significant weight gain was considered to be due to medication, if the medication use coincided with the weight gain, the medication was recognized as potentially obesogenic and if there was no other more likely cause.

### 2.1 Statistical analysis

Statistical analysis was performed using Stata IC Version 14 (StataCorp LLC, College Station). Descriptive data are reported as mean ± standard deviation or median (IQR), as appropriate. Normality was assessed graphically. Means were compared using *t*‐test or ANOVA, as appropriate. Medians were compared using the Wilcoxon rank sum test. Proportions were compared using Fisher Exact test. Cox proportional hazard modeling was used to explore risk factors for the occurrence of the renal endpoint and mortality endpoint. Univariate predictors with a *p* value < 0.25 were included in multivariate Cox modeling. Independent variables were removed stepwise if they were not statistically significant. The assumption of proportional hazards for Cox regression was assessed graphically using log‐log plots for dichotomous univariate predictors and by testing the normal distribution of Schoenfeld residuals for the final multivariate equation. All *p*‐values were calculated as 2‐sided values with statistical significance set at a *p*‐value <0.05.

### 2.2 Ethics statement

Ethics approval was obtained through the ACT Health Human Research Ethics Committee (2022/ETH02130). Due to the nature of the study being a retrospective review of de‐identified data, patient consent was not required.

## 3 RESULTS

On 20 March 2017, 311 patients met the inclusion criteria. One patient was excluded as they subsequently underwent two consecutive kidney transplants after 20 March 2017, with the first transplant failing within 1 month of implantation. The final data set contained 310 individuals (Table 1 Baseline demographics). Baseline eGFR data were missing for 3 patients and their CKD stage was categorized on the basis of the CKD stage recorded in the clinical record. Median age was similar between patients with CKD Stage 3 and 4, but the median age of those with stage 5 CKD was lower than those with less severe CKD (*p* = 0.0002). The median follow‐up period was 4.4 (2.4–5.5) years and 65 patients (21%) were lost to follow‐up with the renal unit. Reasons for loss to follow‐up were discharge 28 (9%), transfer to another hospital 12 (4%) and non‐attendance at clinic 25 (8%).

**TABLE 1:** Baseline data.

Median age in years	Entire Cohort: 75.0, (67.2–80.3)	CKD Stage 3: 75.2 (68.5–79.5)
	CKD stage 4: 75.8 (67.8–81.0)	CKD stage 5: 68.9 (54.7–74.7)
Sex	119 (38%) female	191 (62%) male
CKD stage 3	58 (19%)	
CKD stage 4	210 (68%)	
CKD stage 5 not on dialysis	42 (14%)	
CKD cause (%)	Diabetic kidney disease 164 (53)	Unknown 58 (19)
	Glomerulonephritis 25 (8)	Hypertension 20 (6)
	Obstruction 8 (3)	Other 35 (11)
Systolic BP (*n* = 309)	140.4 ± 17.8 mmHg	
Diastolic BP (*n* = 309)	74.1 ± 11.1 mmHg	
Stage 3 albuminuria (*n* = 252)	115 (46%)	
Mean eGFR (*n* = 307)	23.8 ± 8.4 mL/min/1.73 m^2^	
Median weight	87.4 kg (75.7–99.7)	
Median BMI	31.0 Kg/m^2^ (28.4–35.3)	
Resident in Canberra	190 (61%)	

### 3.1 Weight changes during follow‐up

The mean weight change to the final follow‐up across the entire cohort was −2.8 ± 8.6% (*n* = 283). 98 patients (34.6%) patients lost ≥5% weight and 42 (14.8%) patients gained ≥5% weight. The mean percentage weight change at the final follow‐up was similar across different CKD stages (*p* value for difference = 0.26). Individual weight changes by CKD Stage are shown in Figure S1. There was no evidence of a relationship between baseline eGFR and subsequent weight changes for the group as a whole or for those with baseline eGFR<35 mL/min/1.73 m^2^. During the first year of follow‐up, mean weight change was −0.70 ± 6.44%. 42 patients (21.3% of the 197 with available data) lost ≥5% body weight and 23 (11.7%) gained ≥5% body weight.

During the first 12 months of follow‐up, the most common reason for a clinically significant weight loss was diuresis and intercurrent illness (52%) with deliberate attempts at losing fat mass responsible for weight loss in only 3 (7%) patients. In 17 patients (40%), the reason for weight loss at 12 months was uncertain. The reason for clinically significant weight gain during the first 12 months was attributable to obesogenic drug prescription (insulin or glucocorticoids) in 2 patients (9%), fluid retention in 7 patients (30%) and unknown in 14 patients (61%). No patient appeared to have deliberately gained weight.

### 3.2 Mortality outcome

Loss to follow‐up with the renal unit did not necessarily preclude a collection of mortality data; therefore, no more than 54 patients (17%) had uncertain mortality status at the end of the study. As at 30 September 2022, 128 patients (41.3%) had died, of whom 11 died after being otherwise lost to follow‐up. Mortality was similar across the different baseline CKD stages, *p* value for difference = 0.5 (see Table 2). BMI at baseline did not predict subsequent death but age, ≥5% weight loss and ≥5% weight gain at 12 months were associated with a higher risk of subsequent death. Univariate and multivariate Cox regression predictors for death after the 1‐year weight measurement are shown in Table 3. Limiting the analysis to patients where the reason for the significant weight change was unknown did not materially alter these findings (see Table S1).

**TABLE 2:** Mortality and renal endpoint by chronic kidney disease stage (%).

Baseline CKD stage	Dialysis	Renal endpoint	Died	Died without reaching	
				Dialysis	Renal endpoint
Stage 3 *n* = 58	9 (15.5)	15 (25.9)	20 (34.5)	18 (31.0)	16 (27.6)
Stage 4 *n* = 210	60 (28.6)	75 (35.7)	91 (43.3)	61 (29.0)	56 (26.7)
Stage 5 *n* = 42	33 (78.6)	36 (85.7)	17 (40.5)	4 (9.5)	3 (7.1)

**TABLE 3:** Cox regression for death after 12‐month weight measurement.

	Univariate HR	Univariate CI	Univariate p	**Multivariate HR** ^b^	Multivariate CI	Multivariate *p*
Age	1.06	1.03–1.09	<0.0005	1.06	1.03–1.09	<0.0005
Diastolic BP	0.98	0.96–1.00	0.12			
≥5% weight loss^a^	1.93	1.16–3.21	0.08	2.74	1.61–4.67	<0.0005
≥5% weight gain^a^	1.99	1.07–3.70	0.03	2.67	1.40–5.09	0.003

^a^ Weight change from baseline to 12‐month follow‐up.

^b^ The final multivariate model adjusted for Age at baseline and clinically significant weight change only. No other variables retained statistical significance in the multivariate model.

### 3.3 Renal outcome

The renal endpoint occurred in 126 patients (40.7%) by study end (see Table 2). 5 patients underwent a kidney transplant after commencing dialysis and 3 had a pre‐emptive kidney transplant. On multivariable Cox regression, after excluding those with Stage 5 CKD at baseline, Stage 3 albuminuria, ≥5% weight loss and ≥5% weight gain during the first 12 months were associated with a higher subsequent risk of the renal endpoint (see Table 4). If the analysis was limited to those where the reason for clinically significant 1‐year weight change was unknown, then only Stage 3 albuminuria at baseline remained in the multivariate model (see Table S2).

**TABLE 4:** Cox regression for renal endpoint after 12‐month weight measurement in stage 3 or 4 chronic kidney disease.

	Univariate HR	Univariate CI	Univariate p	Multivariate HR^a^	Multivariate CI	Multivariate p
Age	0.98	0.96–1.00	0.11			
Female sex	0.57	0.33–0.99	0.05			
Stage 3 albuminuria	3.74	2.10–6.34	<0.0005	3.46	2.03–5.91	<0.0005
Stage 3 CKD	0.65	0.35–1.22	0.18			
≥5% weight loss to 12 months	2.42	1.35–4.33	0.003	2.51	1.35–4.67	0.004
≥5% weight gain to 12 months	1.71	0.81–3.58	0.16	2.20	1.015–4.78	0.05

^a^ The final multivariate model included Stage 3 albuminuria at baseline and clinically significant weight change at 12 months only. No other variables retained statistical significance in the final model.

## 4 DISCUSSION

This study was performed in an unselected group of 310 patients with moderate to severe non‐dialysis CKD and overweight or obesity. A significant proportion experienced clinically significant weight changes, but because these changes were balanced, the mean weight for the group fell only modestly during follow‐up. There was no association between baseline severity of CKD and subsequent weight change. This finding is in contrast to earlier publications^11^, ^13^ which found that, below an eGFR of 30–35 mL/min/1.73 m^2^, participants tended to lose weight. This is likely due to the different methodology used in those studies. Both earlier publications reported modeled weight changes after the within study eGFR dropped below 30–35 mL/min/1.73 m^2^ and both reported weight change as an annualized figure. A ≥ ‐5% annualized weight change over the median 4.4 years of follow‐up in our study corresponds to 22% total weight loss. In contrast, our analysis used the measured weight change within 180 days of 1 year follow‐up and the eGFR at baseline. The authors feel that this approach is easier to incorporate into clinical practice as it focusses on clinically relevant weight change over 1 year, rather than potentially including patients with less weight loss over a shorter period of time.

**FIGURE 1:**
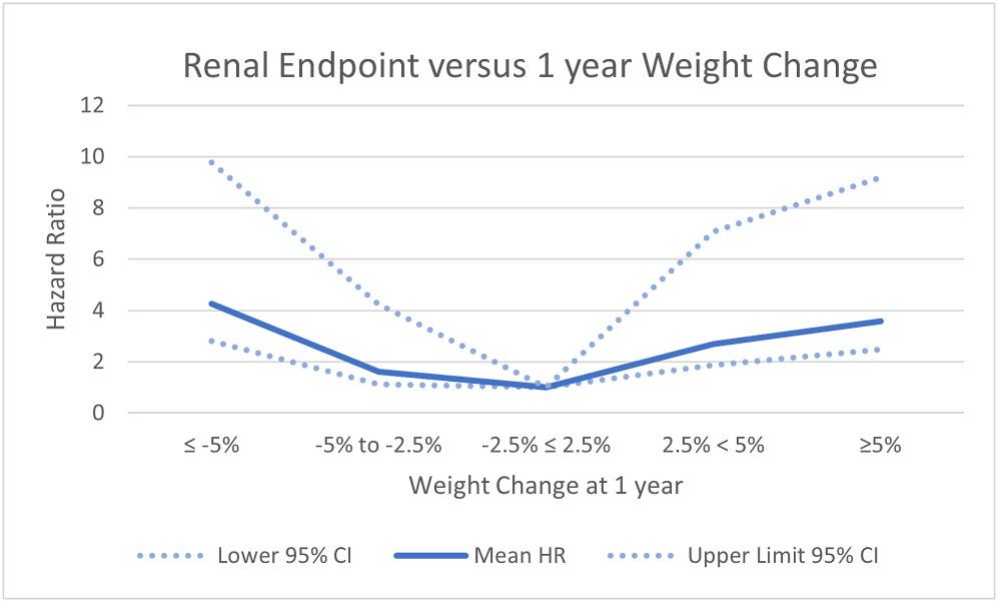
Renal endpoint versus weight change at 1 year.

**FIGURE 2:**
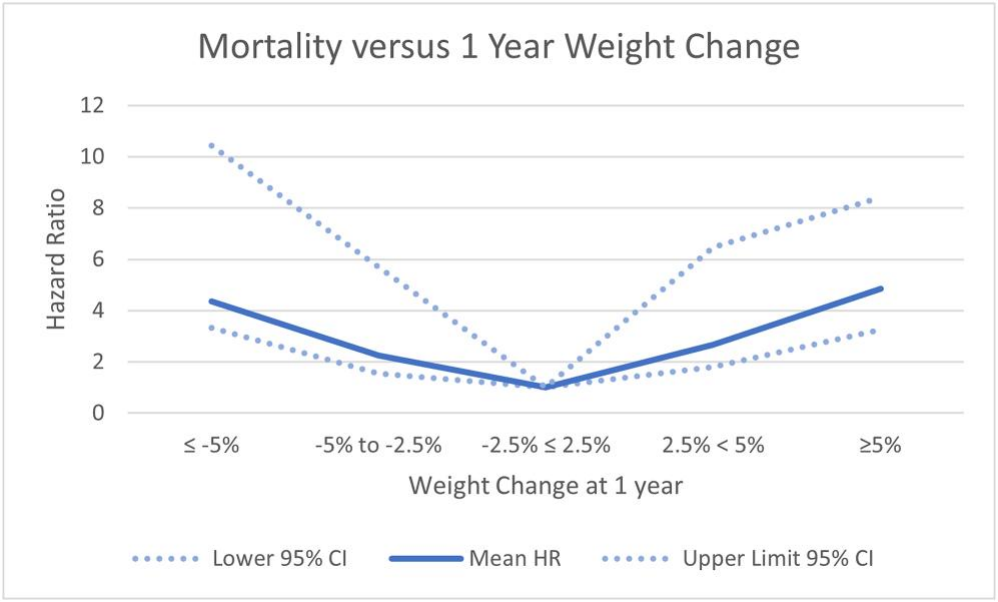
Mortality endpoint versus weight change at 1 year.

The authors found a U‐shaped association between clinically significant weight change during the first year and death or the renal endpoint. This is shown in Figures 1 and 2, where the weight change of −2.5 to +2.5% is the reference point. Excluding patients with clinically apparent reasons for the weight change from the analysis did not materially alter these associations. Previous publications examining weight change and mortality have found either that weight loss of >5%/year prior to dialysis initiation was associated with higher mortality^11^ or found a U shaped relationship between weight loss and adverse outcomes, which is similar to the finding in our study.^12^ A strength of our study was the ability to limit analysis to patients where the cause of weight loss was not clinically apparent. Noting that weight change increases the risk of death or of renal endpoints is not useful if the weight change has been confounded by a clear cause, such as the development of a severe intercurrent medical illness.

Unfortunately, confounding variables are still a significant issue for our study as they are across other observational studies in this area. This is because, in the absence of a deliberate attempt by the patient to gain or lose weight, the weight change likely represents a surrogate marker for other adverse changes in health and may not, itself, be on the causal chain for death. Successful, deliberate attempts to gain or lose weight appear to be rare in the CKD population (3 out of 65 patients). This emphasizes the risk of confounding in relying on observational studies to guide weight loss/gain advice to patients with moderate to severe CKD and overweight or obesity. The impact of deliberate weight loss cannot be reliably assessed in only 3 patients and our study therefore provides no information on the risks or benefits of intentional weight loss in CKD.

Until results from randomized, controlled, intervention studies targeting the weight change in the CKD population are available, clinicians must rely on clinical judgment to guide weight change advice in this population. Observational data on risks and benefits are significantly confounded and cannot be relied upon.

## AUTHOR CONTRIBUTIONS

Richard Singer collected and analyzed the data and wrote the first draft of the manuscript. Hsin‐Chia Huang and Richard Singer designed the study and reviewed the final manuscript.

## CONFLICT OF INTEREST STATEMENT

The authors declare no conflicts of interest.

## Supporting information

Supporting Information S1
