# Supplementary material for: Weight change in chronic kidney disease: Association with mortality and kidney function
Source: Obes Sci Pract. 2023 Nov 22;10(1):e723. doi: 10.1002/osp4.723 (PMC10804345; doi:10.1002/osp4.723)

**Title:**

Weight change in chronic kidney disease: association with mortality and kidney function

**Authors:**

Richard Singer^1, 2^ Hsin-Chia Huang^2, 3^

^1^ Canberra Health Services, Renal Unit. Garran, ACT, Australia.

^2^ Australian National University, School of Medicine. Acton, ACT, Australia

^3^ Canberra Health Services, Respiratory and Sleep Medicine, Garran ACT, Australia

**Corresponding Author Contact Information**

Renal Unit, Canberra Hospital. PO Box 11, Woden ACT 2626, Australia

Email: [Richard.singer@act.gov.au](mailto:Richard.singer@act.gov.au)

Phone: 61251243359

Fax: 61251247063

Table S1: Cox Regression for Death after 12-month weight measurement, where the cause of significant weight change was unknown

|  | Univariate HR | Univariate CI | Univariate p | Multivariate HR | Multivariate CI | Multivariate P |
| --- | --- | --- | --- | --- | --- | --- |
| Age | 1.06 | 1.03 to 1.09 | <0.0005 | 1.06 | 1.02 to 1.09 | 0.001 |
| Diastolic BP | 0.98 | 0.96 to 1.00 | 0.12 |  |  |  |
| >=5% weight loss to 12 months if cause unknown | 1.56 | 0.71 to 3.46 | 0.27 | 2.24 | 0.99 to 5.04 | 0.05 |
| >=5%weight gain to 12 months if cause unknown | 2.00 | 0.90 to 4.42 | 0.09 | 2.55 | 1.14 to 5.74 | 0.02 |

Table S2: Cox Regression for the Renal Endpoint after 12-month weight measurement, where the cause for significant weight change was unknown.

|  | Univariate HR | Univariate CI | Univariate p | Multivariate HR | Multivariate CI | Multivariate P |
| --- | --- | --- | --- | --- | --- | --- |
| Age | 0.98 | 0.96 to 1.00 | 0.11 |  |  |  |
| Female sex | 0.57 | 0.33 to 0.99 | 0.05 |  |  |  |
| Stage 3 albuminuria | 3.74 | 2.10 to 6.34 | <0.0005 | 3.74 | 2.10 to 6.34 | <0.0005 |
| Stage 3 CKD | 0.65 | 0.35 to 1.22 | 0.18 |  |  |  |
| >=5% weight loss to 12 months if cause unknown | 2.14 | 1.00 to 4.58 | 0.05 |  |  |  |

Figure S1: Percentage Weight Change from Baseline by CKD Stage


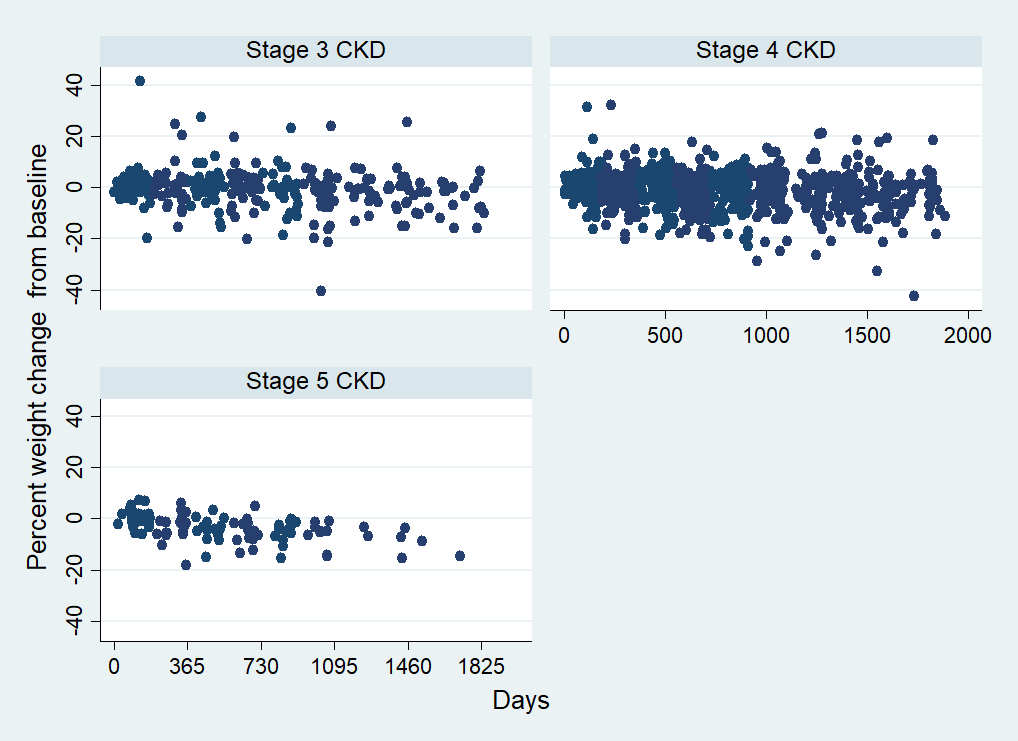

Supplement: Supplementary file 1 — Supporting Information S1 [file OSP4-10-e723-s001.docx]
